# Adrenal and periadrenal schwannoma: histological, molecular and clinical characterization of an institutional case series

**DOI:** 10.1007/s12020-023-03463-y

**Published:** 2023-08-03

**Authors:** Adam Stenman, Henrik Falhammar, Jan Zedenius, C. Christofer Juhlin

**Affiliations:** 1 Department of Molecular Medicine and Surgery, Karolinska Institutet, Stockholm, Sweden; 2 Department of Breast, Endocrine Tumors and Sarcoma, Karolinska University Hospital Solna, Stockholm, Sweden; 3 Department of Endocrinology, Karolinska University Hospital Solna, Stockholm, Sweden; 4 Department of Oncology-Pathology, Karolinska Institutet, Stockholm, Sweden; 5 Department of Pathology and Cancer Diagnostics, Karolinska University Hospital Solna, Stockholm, Sweden

**Keywords:** Adrenal, Schwannoma, Molecular, Radiology, Pathology, Outcome

## Abstract

**Purpose:**

Adrenal schwannoma (AS) and periadrenal schwannoma (PAS) are exceedingly rare Schwann cell tumors that develop from the adrenal medulla and periadrenal peripheral nerves respectively. The underlying genetic events are elusive.

**Methods:**

We searched our institutional database for AS/PAS cases and reviewed the histology and clinical outcome. Comprehensive molecular work-up was performed.

**Results:**

We found reports of 4 AS/PAS cases diagnosed between 1992 and 2022 among the 1248 adrenal lesions submitted for histopathology during the same time period (0.32%). Two patients were male, two were female, and the age span was 59–80 years. Median size was 70 mm (range 50–100 mm), and from a radiology perspective, the lesions were initially suspected of malignant lesions originating from either adrenals or kidneys. Hormonal analyses were normal in all cases. Histologically, three cases were annotated as cellular AS or PAS, and one case was annotated as microcystic AS. Molecular characterization using focused next-generation sequencing did not identify *SMARCB1* or *NF2* mutations, alterations previously associated to schwannoma at other anatomical sites. The postoperative period was without complications for all patients, and follow-up did not show any signs of relapse or metastatic disease.

**Conclusion:**

AS/PAS are rare neoplasms that are most often benign, and the molecular etiology is most likely not related to mutations in established schwannoma-related genes. Since these tumors may be misinterpreted as malignant, knowledge of this entity is essential for radiologists, endocrinologists, surgeons and pathologists.

## Introduction

Rare neoplasia of the adrenal gland such as ganglioneuroma, ganglioneuroblastoma, neuroblastoma, schwannoma and malignant peripheral nerve sheath tumors may arise as stand-alone entities or as composite tumors intermingled with pheochromocytoma. Whereas the definitions of some of these lesions have been widely accepted, the histological, molecular and clinical parameters of these entities are somewhat obscure.

Schwannoma is a peripheral nerve sheath tumor originating from Schwann cells. Although reported in a wide variety of ages, the incidence seems to be highest in middle-aged patients. Schwannomas are mostly sporadic tumors arising predominantly on the limbs or in the head and neck region, but cases have been reported for many parenchymatous organs as well as the retroperitoneum [1–3]. Subsets of cases may develop as part of an underlying syndrome, such as neurofibromatosis type 2 (NF2), Carney complex and familial schwannomatosis [4–6]. The vast majority of tumors are benign and patient outcome is excellent. From a histological context, schwannomas are subdivided based on their growth patterns; including conventional, ancient, cellular, plexiform, epithelioid and microcystic subtypes [7–9]. Malignant transformation is exceedingly rare and may be related to previous radiation therapy [10, 11]. Subsets of cases have been reported to display inactivation of the *NF2* and/or *SMARCB1* tumor suppressor genes [12–14].

Adrenal schwannoma (AS) is defined as a schwannoma arising in the adrenal medulla. If the schwannoma develops in close proximity to the adrenal gland, the suggested terminology is periadrenal schwannoma (PAS). AS/PAS are rare lesions, with less than 200 cases published to date [15]. Notably, the bulk of scientific descriptions is derived from single-institution case series, and extensive molecular characterization of these entities is lacking. Given the lack of clinical, histological and genetic data, we sought to describe institutional cases from our department including findings from imaging, histopathology, molecular work-up and follow-up data.

## Materials and methods

In total, we found three AS cases and one PAS case during 1992 to 2022 (with 1992 as the starting year given the earliest inputs in our electronic search system). We used the Systematized Nomenclature of Medicine (SNOMED) code T93*** to retrieve all 1248 histopathologically diagnosed adrenal lesions during these years, specifically looking for M956** (including “neurilemmoma”, the SNOMED assigned term for schwannoma). This search function generated four cases corresponding to 0.32% of diagnosed cases. The patient charts and pathology reports of each case were reviewed. As the quality of the staining may deteriorate over time, blocks were recut and restained for hematoxylin-eosin (H&E). We defined AS as a schwannoma arising within the adrenal medulla, and PAS as schwannoma arising in peripheral nerves in close association to the adrenal gland. We considered SOX10, S100 and GFAP immunohistochemistry essential to the diagnosis, and therefore stained any case lacking one or several of these markers in their original report. Furthermore, all four cases were subjected to molecular characterization using an established next-generation sequencing (NGS) pipeline (Oncomine Childhood Cancer, Thermo-Fisher Scientific, Waltham, MA, USA) used in clinical routine at the Department of Pathology and Cancer Diagnostics, Karolinska University Hospital, Stockholm, Sweden. In brief, this platform interrogates DNA and RNA extracted from formalin-fixated paraffin embedded tissue for mutations in 126 genes (full exon coverage of 44 genes and hotspot coverage of additional 82 genes) and >1700 gene fusion variants in 88 genes. The methodology regarding nucleic acid extraction and sequencing has been previously published [16].

## Results

The overall clinical, histopathological and immunohistochemical findings are presented in Table 1. Below is a detailed description of all four cases.

**Table 1:** Clinical and histological details of the institutional adrenal/periadrenal schwannoma cohort

Case no.	Sex	Age at surgery	Preop. imaging	Attenuation (HU)	18F-FDG uptake	Tumor size (mm)	Tumor weight (g)	Gross cut surface	Histological subtype
**1**	M	80	CT	n.p.	n.p	100	750	White to yellow	Microcystic AS
**2**	F	66	CT and PET	11	Peripheral	105	360	White to yellow^a^	Cellular PAS
**3**	F	59	CT	28	n.p	53	113	White to yellow	Cellular AS
**4**	M	72	CT and PET	25	Enhanced	75	241	White	Cellular AS

Case no.	Necrosis	Mitotic activity (per 10 HPF)	S100	SOX10	GFAP	Pan-CK	Ki-67 index	Follow-up (months)	Outcome
**1**	No	0	+	+	+	-	1%	>200	DOC
**2**	No	1	+	+	+	-	5%	78	AWOD
**3**	No	0	+	+	+	-	13%	22	AWOD
**4**	No	6	+	+	+	-	14%	1	AWOD

*M* male, *F* female, *CT* computerized tomography, *PET* positron emission tomography, *HU* Houndsfield units, *18F-FDG* Flourine-18 fluorodeoxyglucose, *mm* millimeter, *g* gram, *AS* adrenal schwannoma, *PAS* periadrenal schwannoma, *HPF* high-power fields, *n.p.* not performed, *Pan-CK* pan-cytokeratin cocktail, *DOC* dead of other causes, *AWOD* alive without disease

^a^Degeneration was also seen

### Case 1

The patient was an 80-year-old male of Middle Eastern origin, lacking family history indicative of adrenal tumors or other cancers. A computerized tomography (CT) scan performed previously in his home country revealed a cystic expansivity measuring 100 mm suspicious of originating from the pancreatic tail. He presented at our hospital with diffuse abdominal pain. A gastroscopy was performed, but no significant findings were reported. Repeated CT scan showed an unaltered lesion compared with the previous examination, but the radiologists indicated a possible left renal origin. No lesions suspicious for metastases were detected. He underwent open surgery three weeks later, including *en bloc* removal of the retroperitoneal, left-sided tumor along with the spleen. The removed lesion weighed 750 g, including 300 mL of cystic fluid, measuring 100 × 100 × 100 mm. Histology was consistent with a tumor in direct association to the adrenal medulla, composed of bland spindle cells against a myxoid background, with scattered microcystic structures. Immunohistochemistry revealed positivity to SOX10, S100, GFAP and vimentin while showing negative stain for pan-cytokeratin, and the Ki-67 proliferation index was 1% (Table 1). The final diagnosis was microcystic AS without signs of malignancy. Focused NGS screening of somatic DNA did not pinpoint any pathogenic variants or gene fusions. The postoperative period was without any complications. Follow-up did not reveal any signs of relapse and the patient died from non-related disease 19 years after the AS diagnosis.

### Case 2

**Fig. 1:**
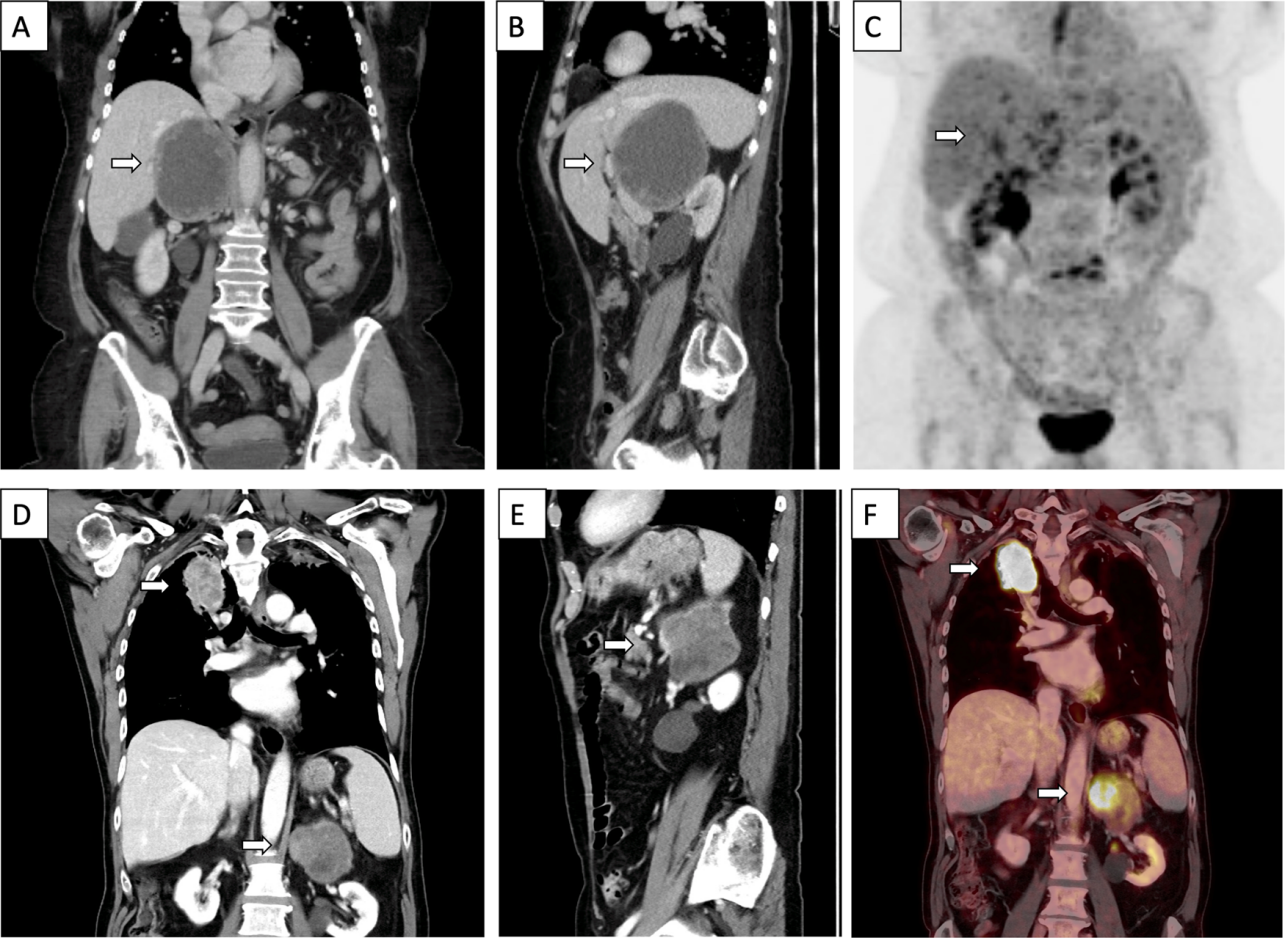
Radiological images of adrenal and periadrenal schwannoma. Depicted here are images of case 2 (**A**–**C**) and 4 (**D**–**F**). White arrows indicate the tumors in all images. **A** Coronal view of computerized tomography (CT) scan, showing the incidentally found 9 cm lesion cranial to the right kidney, suspected of originating from the right adrenal gland. **B** Sagittal view of the same CT scan as in **A**. **C** Coronal view of flourine-18 fluorodeoxyglucose positron emission tomography/computed tomography (^18^F-FDG PET/CT) containing the suspected adrenal mass with an assumed necrotic center and viable periphery. **D** Coronal view of CT scan showing both the 50 × 50 mm mass in the right upper lobe of the lung, and the 75 × 50 mm lesion in the left adrenal. **E** Sagittal view of the adrenal lesion described in **D**. **F** Coronal view of ^18^F-FDG PET/CT showing ^18^F-FDG uptake in both the pulmonary mass and in the left adrenal tumor

**Fig. 2:**
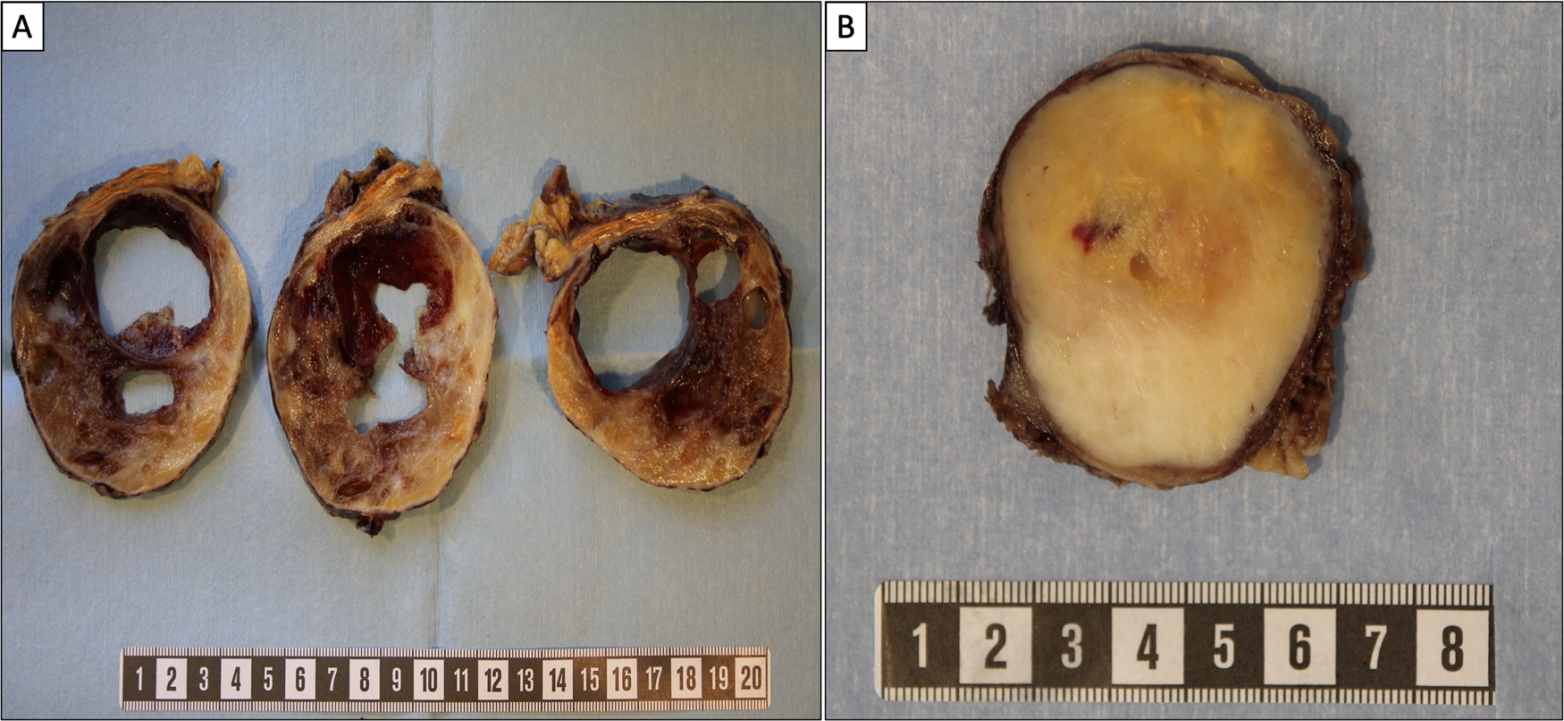
Macroscopic findings of adrenal and periadrenal schwannoma. **A** The 105 mm periadrenal schwannoma of case 2 exhibited central degeneration and a viable tumor periphery with white to yellow cut surface. The gross findings were reminiscent of a cystic pheochromocytoma. Note the adjacent adrenal gland superior to the tumor. **B** The 53 mm adrenal schwannoma of case 3 with a solid, circumscribed mass with a white to yellow coloring. The texture was rubbery/elastic and different from adrenal cortical tumors that otherwise may have a similar color tone

The patient was a 66-year-old female of Swedish ethnicity without family history suggestive of adrenal tumors or other cancers. She presented at the emergency department with dyspnea, and a CT scan was performed to rule out pulmonary embolism. Incidentally, a 9 cm lesion with attenuation of 11 Hounsfield Units (HU), (the tumor rim showed an attenuation of 30 HU), was seen just cranial to the right kidney, suspected of originating from the right adrenal gland (Fig. 1A, B). A gastroscopy was performed, revealing esophagitis. Flourine-18 fluorodeoxyglucose positron emission tomography/computed tomography (^18^F-FDG PET/CT) showed an adrenal mass with an assumed necrotic center and viable periphery (Fig. 1C). She was discussed at a multidisciplinary tumor board conference where the CT scan was reviewed, and surgery was recommended. Hormonal workup was normal with normal plasma metanephrines and cortisol levels, and the patient was normotensive. She underwent open surgery four weeks later, including removal of the right adrenal gland together with para-caval lymph nodes. The removed lesion weighed 360 g, measuring 105 × 50 × 50 mm. During grossing, the tumor was juxta-positioned to the adrenal gland and found to exhibit central degeneration upon grossing (Fig. 2A). Histology of the peripheral parts of the lesion demonstrated a cellular spindle cell tumor with central degeneration, positive for SOX10, S100 and GFAP (Table 1). The final diagnosis was a cellular PAS without signs of malignancy, supported by the finding of six lymph nodes free of metastatic components. Focused NGS screening of the tumor tissue could not identify any pathogenic variants or gene fusions. The postoperative period was without any complications. Follow-up did not reveal any signs of relapse and she is currently alive and well six years after diagnosis.

### Case 3

The patient was a 59-year-old female of Hispanic origin with a history of psoriatic arthritis and fibromyalgia who previously underwent appendectomy for appendicitis. She lacked family history suggestive of adrenal tumors or other cancers. She presented at the emergency department with abdominal pain, and a CT scan was performed to rule out nephrolithiasis. A 50 mm hyper-vascular lesion was seen cranially to the left adrenal vein, with attenuation of 28 HU, suspected of originating from the left adrenal gland. Two enlarged lymph nodes were seen caudally to the left renal artery. Hormonal workup was normal, including plasma metanephrines, the cortisol level post 1 mg overnight dexamethasone suppression test and aldosterone/renin ratio. No hypertension was seen. She underwent open surgery two weeks later, including removal of the tumor and the enlarged lymph nodes. The removed lesion weighed 113 g, measuring 53 × 50 × 50 mm (Fig. 2B). The histopathological diagnosis was cellular adrenal schwannoma arising in the adrenal medulla, positive for SOX10, S100 and GFAP (Table 1). Nine regional lymph nodes free of metastatic components were also reported. No pathogenic mutations or fusions were detected using a focused NGS screening of tumor DNA. The postoperative period was without any complications. Follow-up did not reveal any signs of relapse and she is currently alive and well two years after diagnosis.

### Case 4

**Fig. 3:**
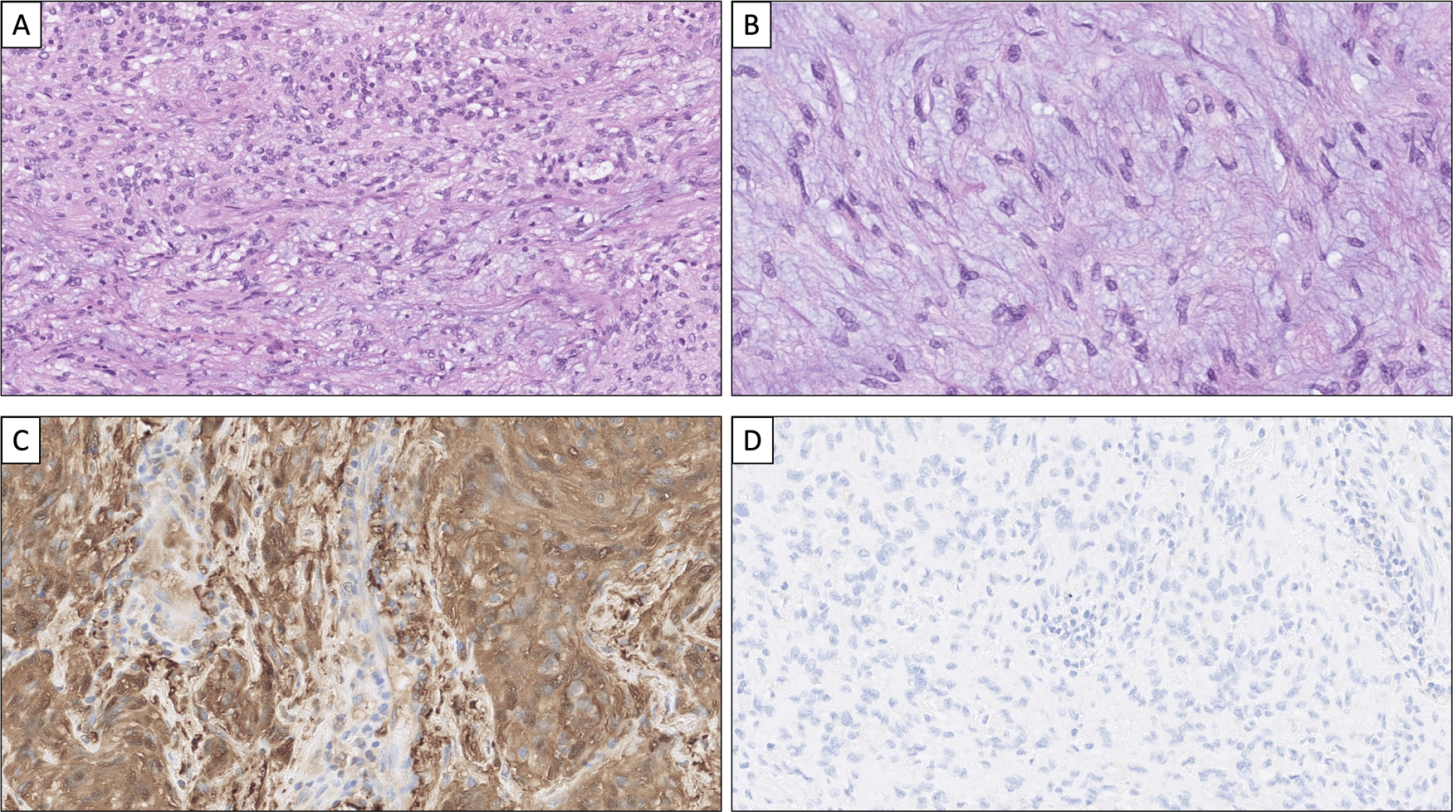
Histological hallmarks of adrenal schwannoma. **A** Cellular adrenal schwannoma diagnosed in patient 4, composed of bland spindle cells without nuclear atypia. Verocay bodies are absent in this schwannoma subtype. **B** Areas with myxoid stroma are occasionally seen even in hypercellular areas. **C** Tumor cells stain intensely for S100. Note the endothelial cells acting as internal negative controls. **D** Adrenal and periadrenal schwannomas are negative for pan-cytokeratin (as in this case stained for AE1/AE3)

The patient was a 72-year-old male of Swedish ethnicity, ex-smoker and previously treated for tuberculosis. He had no family history indicative of adrenal tumors or other cancers. He presented at the emergency department due to general deterioration, and a CT scan was performed. Two lesions were found, a 50 × 50 mm mass in the right upper lobe of the lung, and a 75 × 50 mm lesion in the left adrenal (attenuation 25 HU), the latter suspected initially to be metastatic lung carcinoma (Fig. 1D, E). An ^18^F-FDG PET/CT showed high uptake in both the pulmonary mass and in the left adrenal tumor (Fig. 1F). Cytological examination of the lung lesion was performed followed by a bronchoscopy-guided biopsy, and a diagnosis of non-small-cell lung adenocarcinoma was rendered. The hormonal workup was normal (including etanephrines, cortisol, ACTH and aldosterone/renin ratio). No hypertension was seen. The patient was discussed at a multidisciplinary tumor board conference, in which adrenal surgery was recommended. The patient underwent laparoscopic transabdominal surgery two weeks later, in which the left-sided adrenal tumor was removed. The excised lesion weighed 241 g, measuring 75 × 60 × 50 mm. Histopathology was consistent with an adrenomedullary tumor characterized by hypercellular areas with spindle cells positive for SOX10, S100 and GFAP, and hence metastatic lung carcinoma could be excluded (Fig. 3). The final diagnosis was cellular AS. NGS analyses did not pinpoint any pathogenic mutations or gene fusion events. The postoperative period was without any complications, and he is now treated for his lung adenocarcinoma.

## Discussion

AS and PAS are infrequently encountered lesions in the clinical setting, and hence the data regarding histological attributes, molecular drivers and clinical presentation is limited. A recent meta-analysis from 2022 identified 169 published AS cases [15]. The majority of AS and PAS present as incidentalomas either due to radiological investigations or due to symptoms of mass effect. The typical AS patient is middle-aged, and exhibits a unilateral, non-functioning adrenal mass with a median size of 60 mm [15]. AS usually presents as a solitary and encapsulated mass with a homogenous appearance on a CT scan, and the mean attenuation is slightly over 30 HU, thus most often making AS readily distinguishable from adrenal cortical adenoma and myelolipoma [15, 17]. The attenuation of the tumors in our series ranged from 11–28 HU. Case 3 presented with a hypervascular lesion on imaging, thus radiologically suspicious for pheochromocytoma or adrenocortical carcinoma (ACC). As AS and PAS are large lesions that often present with cystic degeneration and/or necrosis, non-functioning ACCs could constitute a potential differential diagnosis in the radiological work-up. It is also worth stressing the fact that ACCs might be more common than AS and PAS in the general populace, thereby also highlighting the clinical need to distinguish these entities rapidly. Other imaging techniques, such as ^18^F-FDG PET/CT, are rarely used in the diagnostic work-up of AS and PAS, and the few reported cases investigated with this modality show a widespread distribution in terms of uptake, possibly due to differences in histological subtyping and cellular composition [18]. In our case series, the PAS of patient 2 had peripheral ^18^F-FDG uptake only, most likely correlating to the wide-spread central degeneration of tumor tissue, while the ^18^F-FDG uptake in patient 4 was enhanced.

Histologically, most AS and PAS cases are described as either conventional or cellular, while other histological subtypes are rare. A conventional AS/PAS exhibits hypercellular areas (denoted as “Antoni A”) intermingled with palisading spindle cells (Verocay bodies) as well as various amounts of hypocellular areas (“Antoni B”). Cellular AS/PAS cases, however, usually lack Antoni B areas and the Verocay bodies that are seen in conventional AS [19]. In our series, histological subtyping revealed that 3 cases were subtyped as cellular AS or PAS, and a single case was annotated as microcystic AS. Of note, the hypercellular appearance and the lack of loosely populated Antoni B areas and classic Verocay bodies led to extensive immunohistochemical analyses of these three cases, and a number of markers were assayed in order to rule out neurofibroma (CD34), leiomyosarcoma (desmin), angiosarcoma (CD34 and ERG), metastatic GIST (c-kit, DOG1), malignant melanoma (Melan A), pheochromocytoma (chromogranin A) as well as adrenal cortical tumors (SF1, Alpha-inhibin). It seems appropriate to include a wide variety of differential diagnoses in the clinical work-up of AS and PAS, given the rarity of the disease and the existing palette of malignant lesions with morphological similarities [20, 21]. In our experience, typical morphology in addition to positive staining for SOX10, S100 and GFAP is diagnostic for AS/PAS.

The focused NGS screening did not detect any pathogenic variants or gene fusion events in this small case series. Although *NF2* and *SMARCB1* mutations have been reported in schwannomas from other anatomical sites [12–14], we did not observe these aberrations. Hence, the molecular background of AS/PAS remain elusive, and pan-genomic analyses could be required to identify potential driver events.

As expected, all patients in this series were free of metastatic disease. In fact, only a few cases of AS with malignant transformation have been reported [22, 23]. Two of our cases (cases 3 and 4) exhibited substantially higher Ki-67 indices than cases 1 and 2, but there was no clear-cut correlation between the mitotic activity and this labeling index or any other histological parameter. While Ki-67 has been proposed as a predictive marker for recurrence in vestibular schwannoma, little is known regarding the potential value of this analysis in terms of AS/PAS [24].

We conclude that AS and PAS are rare lesions with a radiologically heterogenous presentation. As the lesions might be misinterpreted as malignant during preoperative workup, increased knowledge of these entities is of importance for radiologists, endocrinologists, surgeons and pathologists alike.
